# Familial Clustering of Venous Thromboembolism – A Danish Nationwide Cohort Study

**DOI:** 10.1371/journal.pone.0169055

**Published:** 2016-12-29

**Authors:** Caroline Sindet-Pedersen, Louise Bruun Oestergaard, Anna Gundlund, Emil Loldrup Fosbøl, Kristian Aasbjerg, Jannik Langtved Pallisgaard, Gunnar Gislason, Christian Torp-Pedersen, Jonas Bjerring Olesen

**Affiliations:** 1 Department of Cardiology, Copenhagen University Hospital Herlev and Gentofte, Hellerup, Denmark; 2 Faculty of Health and Medical Sciences, University of Copenhagen, Copenhagen N, Denmark; 3 Institute of Health, Science and Technology, Aalborg University, Aalborg, Denmark; 4 The Danish Heart Foundation, Copenhagen K, Copenhagen, Denmark; 5 Department of Cardiology, Copenhagen University Hospital Rigshospitalet, Copenhagen Ø, Denmark; 6 Department of Ophthalmology, Aalborg University Hospital, Aalborg, Denmark; 7 The National Institute of Public Health, University of Southern Denmark, Odense, Denmark; Institut d'Investigacions Biomediques de Barcelona, SPAIN

## Abstract

**Background:**

Identification of risk factors for venous thromboembolism (VTE) is of utmost importance to improve current prophylactic regimes and treatment guidelines. The extent to which a family history contributes to the risk of VTE needs further exploration.

**Objectives:**

To examine the relative rate of VTE in first-degree relatives compared with the general population.

**Methods:**

By crosslinking Danish nationwide registries we identified patients with VTE between 1978 and 2012, and their familial relations. The first member in a family to acquire VTE was defined as the proband. All first-degree relatives to probands were followed from the VTE date of the proband and until an event (VTE), death, emigration, 100 year birthday or end of study: 31^st^ of December 2012, whichever came first. The relative rate of VTE was estimated by standardized incidence ratios (SIR) using time-dependent Poisson regression models, with the general population as a fixed reference.

**Results:**

We identified 70,767 children of maternal probands, 66,065 children of paternal probands, and 29,183 siblings to sibling probands. Having a maternal proband or a paternal proband were associated with a significantly increased VTE rate of 2.15 (CI: 2.00–2.30) and 2.06 (CI: 1.92–2.21), respectively. The highest estimate of VTE was observed among siblings (adjusted SIR of 2.60 [CI: 2.38–2.83]). Noteworthy, the rate of VTE increased for all first-degree relatives when the proband was diagnosed with VTE in a young age (≤ 50 years).

**Conclusion:**

A family history of VTE was associated with a significantly increased rate of VTE among first-degree relatives compared with the general population.

## Introduction

Venous thromboembolism (VTE), i.e. deep venous thrombosis (DVT) and pulmonary embolism (PE), poses a great burden for both patients and healthcare systems worldwide. [1–3] VTE affects 1–2 out of 1,000 individuals each year, and is associated with a high risk of morbidity and mortality. [1–6] VTE is a multifactorial disease, possibly caused by an interim of both genetic and environmental risk factors. [7, 8] Genetic risk factors for VTE have been recognized since the 1960s when the antithrombin deficiency was first discovered. [9, 10] As of today, several genetic risk factors have been identified including; factor V Leiden, protein C and, protein S deficiency. [10–13] However, from genetic variation studies, common polymorphisms have been found to only account for 5% of VTE heritability. [9, 14, 15] VTE tend to cluster in families and known thrombophilia’s does not fully explain the increased risk in these individuals. [16, 17] Previous studies have observed an increased risk of VTE among first-degree relatives [17–30] and have revealed that a family history of VTE is an independent risk factor irrespective of known thrombophilia.[17] However, some of these studies were limited by investigating the risk in subpopulations, by their sample size, and by using questionnaires. [23–29] In addition, the studies have been limited by only examining the risk of VTE in either, siblings [20, 21] or parents. [19] In the search for potential genetic risk factors, it is important to characterize the familial risk of VTE in detail. Furthermore, identification of VTE risk factors and thereby high-risk patients is essential to improve primary prophylactic regimes and treatment guidelines. Thus the aim of this study was to provide a thorough description of the risk of VTE among first-degree relatives according to probandship and according to the age at diagnosis of the proband.

## Methods

### Data sources

In this cohort study, we used the Danish nationwide administrative registries, from which it is possible to obtain information on all hospital contacts, all prescribed medicines from pharmacies, and date of birth etc. [31–34] All Danish residents are at birth or migration given a unique identification number, which enables cross linkage between the nationwide Danish registries on an individual level. [31] Information from the Danish National Patient Register includes all in-patients hospital contacts from 1978 and onward, with out-patient and emergency department visits registered from 1995 and onwards. [32] All diagnoses are registered according to the International Classification of Diseases (ICD) and are classified as either a primary or secondary diagnosis. Before 1994 diagnoses were registered according to the 8^th^ version and after 1994 according to the 10^th^ version. [32] From the Danish National Prescription Registry, information on all prescribed dispensed medicines can be obtained from 1995 and onward. [33] The Danish Civil Registration system provides information on date of birth, date of death, sex, emigration and immigration. The Danish Fertility Registry, has since 1954, collected information on familial relations, making it possible to obtain information on parents, children, and siblings.

### Study population

By cross-linking the Danish National Patient Registry with the Civil Registration System, all patients (inpatients and outpatients) in the period of 1978–2012 with a primary or secondary discharge diagnosis of VTE were identified. Through the Danish Fertility Registry familial relations were identified, parents with VTE or the first sibling in a sib-ship with VTE were defined as the proband. First-degree relatives to probands (children of maternal probands, children of paternal probands, and sibling-to-sibling probands) were followed from the probands’ date of VTE, date of birth or immigration whichever came first, and until 100 years of age, emigration, developing VTE, death, or 31^st^ of December 2012. We excluded patients who died before 1^st^ of January 1978, stillborn babies, and adopted children. It was possible for the first-degree relatives to be classified in more than one group, meaning that i.e. a child of a maternal proband could also be classified as a sibling to a sibling proband.

### Covariates

#### Comorbidities

Information regarding comorbidities was achieved from the National Patient Register (ICD- 8 and ICD-10 diagnose codes). These included; stroke, myocardial infarction, atrial fibrillation, ischemic heart disease, vascular disease, chronic heart failure, cancer, chronic kidney disease, liver disease, and chronic obstructive pulmonary disease. The diagnosis of hypertension and diabetes was defined from a diagnosis or from the use of either anti-diabetic drugs or antihypertensive medication (S1 Table).

#### Concomitant medication

Baseline pharmacotherapy of included patients was examined using the Danish Register of Medicinal Product Statistics and included: ADP-inhibitors, calcium antagonists, beta-blockers, diuretics, renin- angiotensin inhibitors, ACE-inhibitors, non-steroidal anti-inflammatory drugs, lipid modifying agents, ulcer medication, antipsychotics, oral anticoagulation therapy, and heparin (S2 Table).

### Statistical analyses

The absolute risk of VTE among first-degree relatives was examined by crude cumulative incidence curves taking into account the competing risk of death. The relative risk of VTE among first-degree relatives was estimated by Poisson regression modeling, where the general population was used as a fixed reference, as has been performed previously.[35, 36] Two Poisson models were constructed; the first model was adjusted for age, sex, and calendar year and the second model was adjusted for age, sex, calendar year, and the aforementioned comorbidities and concomitant medication. All variables were entered as time dependent variables, making it possible to update; calendar year, age, comorbidities, and concomitant pharmacotherapy continuously every 5 years. [35, 36] Results were shown as standardized incidence ratios with 95% confidence intervals (CI). Interaction was examined using an interaction term in the model, and if significant the analyses were stratified where appropriate. All statistical analyses were performed at a 0.05 significance level. Handling of data and statistical analysis was performed using SAS (Statistical Analytical System, version 9.4, SAS Institute, Gary, NC.) and R (version 3.0.2 for Windows, R Foundation for Statistical Computing).

### Subgroup and sensitivity analyses

All first-degree relatives to probands were subdivided according to age of diagnoses of the proband; < 30, 30–49, 50–59, 60–69, and ≥ 70 years of age, and in supplementary analyses, into ≤ 50 and >50 years of age at diagnosis of the proband. To evaluate a potential familial aggregation of VTE, we examined the rate of the condition in individuals having one or more probands previously diagnosed with VTE. Furthermore, an analysis on the risk of VTE among spouses was performed, in order to investigate the influence of potential environmental risk factors. The first spouse in the marriage to be diagnosed with VTE was defined as the proband. An additional sensitivity analysis was performed, where the risk of VTE was investigated in adopted children of mothers and fathers with VTE.

### Ethics

Retrospective registry-based studies do not require approval from the Research Ethics Committee System. The Danish Data Protection Agency had approved use of data for this study (ref.no: 2007-58-0015 / GEH-2014-012 I-Suite no: 02720).

## Results

**Fig 1:**
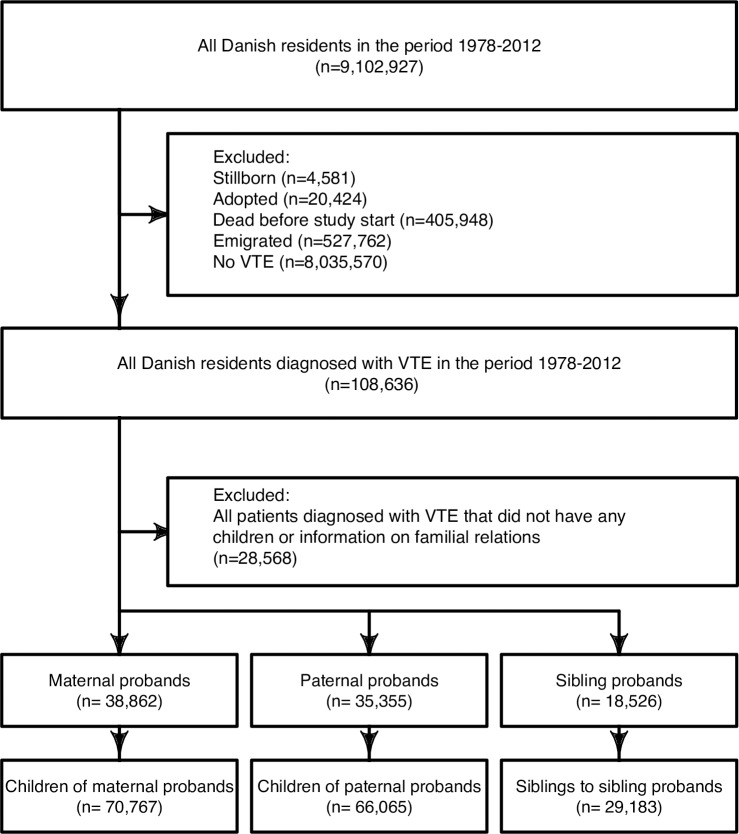
Flowchart over selection process. Study cohort, was compromised of patients discharged with VTE from 1978–2012. The cohort was further divided into probandship, and further into children of maternal and paternal proband and sibling to sibling probands.

From 1978 to 2012, a cumulative number of 9,102,927 Danish residents were identified (Fig 1). Of these 108,636 patients were admitted with a first-time VTE and from these patients, 38,862 patients were identified as maternal probands, 35,355 were identified as paternal probands and 18,156 were identified as sibling probands. A total of 166,615 first-degree relatives to probands were identified, of which: 70,767 were children of maternal probands, 66,065 were children of paternal probands, and 29,183 were sibling-to-sibling probands. Because the first-degree relatives could be classified in more than one group, 160,615 first-degree relatives were identified, when combining children of maternal and paternal proband and siblings to sibling probands.

Baseline characteristics of probands and first-degree relatives are presented in Tables 1 and 2, respectively. The median age was higher among paternal and maternal probands than among sibling probands. Approximately 70% of the probands had DVT and 30% had PE as index event. The maternal and paternal probands had more comorbidity and used more concomitant medication than the sibling probands. The most present comorbidity was hypertension, diabetes, and cancer.

**Table 1:** Baseline characteristics of probands, divided into maternal, paternal and sibling probands.

Variables	Maternal proband N = 38,862	Paternal proband N = 35,355	Sibling proband N = 18,526
**Patient characteristic**			
Median age (IQR)	64 (49–74)	64 (54–73)	39 (31–46)
Males (%)	0 (0)	35,355 (100)	8,467(45.7)
**Index event**			
DVT (%)	26,035 (70.0)	23,977 (67.8)	14,205 (76.6)
PE (%)	12,827 (30.0)	11,378 (32.2)	4,321 (23.4)
**Comorbidities (%)**			
Stroke	2,304 (5.93)	2,529 (7.15)	311 (1.68)
Acute myocardial infarction	1,890 (4.86)	3,346 (9.46)	247 (1.34)
Atrial fibrillation	2,512 (6.46)	3,021 (8.54)	218 (1.18)
Ischemic heart disease	5,082 (13.08)	6,620 (18.72)	691 (3.73)
Vascular disease	1,431 (3.68)	1,635 (4.62)	199 (1.07)
Artery disease	454 (1.17)	4,41 (1.25)	188 (1.01)
Chronic heart failure	3,108 (8.0)	3,446 (9.75)	361 (1.95)
Chronic kidney disease	801 (2.06)	1,150 (3.25)	314 (1.69)
Liver disease	691 (1.78)	716 (2.03)	331 (1.79)
Hypertension	1,717 (30.15)	9,953 (28.15)	1,551 (8.37)
Diabetes mellitus	3,358 (8.64)	3,343 (9.46)	640 (3.46)
Cancer	7,864 (20.24)	6,982 (19.75)	1,305 (7.04)
COPD	3,995 (10.28)	3,470 (9.81)	394 (2.13)
**Concomittant medication**			
ADP-receptor blockers	9,437 (23.76)	9,465 (26.77)	1,058 (5.71)
Aspirin	9,180 (23.62)	9,362 (26.48)	1,057 (5.71)
Diuretics	13,615 (35.03)	8,829 (24.97)	1,931 (10.42)
Loop	9,879 (25.42)	7,097 (20.07)	1,372 (7.42)
Beta-blockers	9,193 (23.66)	7,614 (21.54)	1,867 (10.08)
Calcium channel blockers	8,090 (20.82)	7,364 (20.83)	1,063 (5.74)
Renin-angiotensin system blockers	9,269 (23.85)	8,794 (24.87)	1,423 (7.68)
Vasodilators	30 (0.08)	34 (0.10)	17 (.0.09)
Digoxin	1,798 (4.63)	1,977 (5.59)	82 (0.44)
Oral anticoagulation	2,919 (7.51)	3,442 (9.74)	1,172 (6.33)
Heparin	533 (1.37)	383 (1.08)	288 (1.55)
Statin	5,861 (15.08)	5,715(16.16)	856 (4.62)
Antipsychotics	4,575 (11.77)	2,535 (7.17)	1,822 (9.83)
Non-steroidal anti-inflammatory drugs	28,449 (73.21)	24,020 (67.94)	12,965 (69.98)
Anti-diabetics without insulin	2,272 (5.85)	2,240 (6.34)	449 (2.42)
Anti-diabetics with insulin	111 (2.60)	1,003 (2.84)	323 (1.74)

**Table 2:** Baseline characteristics of first-degree relatives divided into children of maternal or paternal probands and siblings to sibling probands.

Variables	Children of maternal proband N = 70,767	Children of paternal proband N = 66,065	Sibling to sibling N = 29,183
**Patient characteristic**			
Median age (IQR)	35 (21–44)	34 (23–42)	38 (30–45)
Males (%)	37,403 (52.9)	34,848 (52.8)	15,487 (53.1)
**Comorbidities (%)**			
Stroke	286 (0.40)	192 (0.29)	156 (0.53)
Acute myocardial infarction	260 (0.37)	150 (0.23)	152 (0.52)
Atrial fibrillation	215 (0.30)	156 (0.24)	133 (0.46)
Ischemic heart disease	748 (1.06)	492 (0.74)	441 (1.51)
Vascular disease	104 (0.15)	68 (0.10)	73 (0.25)
Artery disease	36 (0.05)	20 (0.03)	16 (0.05)
Chronic heart failure	307 (0.43)	225 (0.34)	153 (0.52)
Chronic kidney disease	210 (0.30)	176 (0.27)	108 (0.37)
Liver disease	245 (0.35)	191 (0.29)	145 (0.50)
Hypertension	2,754 (3.89)	1721 (2.61)	1,420 (4.87)
Diabetes mellitus	1,210 (1.71)	935 (1.42)	641 (2.20)
Cancer	1,101 (1.56)	930 (1.41)	499 (1.71)
COPD	481 (0.68)	341 (0.52)	261 (0.89)
**Concomitant medication**			
ADP-receptor blockers	1,205 (1.70)	798 (1.21)	839 82.60)
Aspirin	1245 (1.76)	830 (1.26)	763 (2.61)
Diuretics	3,179 (4.49)	2092 (3.17)	1,672 (5.73)
Loop	1,043 (1.47)	740 (1.12)	608 (2.08)
Beta-blockers	3,708 (5.24)	2,983 (4.52)	1,894 (6.49)
Calcium channel blockers	1,763 (2.49)	1,129 (1.71)	875 (3.00)
Renin-angiotensin system blockers	2,654 (3.75)	1,667 (2.52)	1,424 (4.88)
Vasodilators	6 (0.01)	6 (0.01)	<3(0.01)
digoxin	80 (0.11)	45 (0.07)	40 (0.14)
Oral anticoagulation	246 (0.35)	168 (0.25)	155 (0.53)
Heparin	69 (0.10)	64 (0.10)	50 (0.19)
Statins	1702 (2.41)	1,030 (1.56)	1,031 (3.53)
Antipsychotics	2,462 (3.48)	1,967 (2.98)	1,376 (4.72)
Non-steroidal anti-inflammatory drugs	31,589 (44.64)	2,8591(43.28)	1,6523 (56.58)
Anti-diabetics without insulin	659 (0.93)	459 (0.69)	437 (1.50)
Anti-diabetics with insulin	552 (0.78)	467 (0.71)	309 (1.06)

Table 2 shows the baseline characteristics of the children of maternal and paternal probands along with sibling-to-sibling probands. The first-degree relatives had less comorbidity and used less concomitant medication than their probands, mainly due to the lower age at inclusion in to the study.

### Risk of VTE

All first-degree relatives were followed for a median time of 5.8 years [inter quartile range (IQR): 2.6–9.8]. In this time period, 1,983 developed VTE at a median age of 42 [IQR: 34–49]. Children of maternal probands were followed for a median time of 5.95 years [IQR: 2.6–10.4], and 823 developed VTE at a median age of 44 [IQR: 36–50] years. Children of paternal probands were followed for 6.7 years [IQR: 3.3–10.4], and 769 developed VTE at a median age of 41 [IQR: 33–48] years. Of the sibling-to-sibling probands, 517 developed VTE at a median age of 43 [IQR: 36–49] years during a median time of follow-up of 4.8 [IQR: 1.9–8.7] years. The Danish general population was followed for a median time of 25.0 years [IQR: 10.8–35.0] and developed VTE at a median age of 67 [IQR: 52–78].

**Fig 2:**
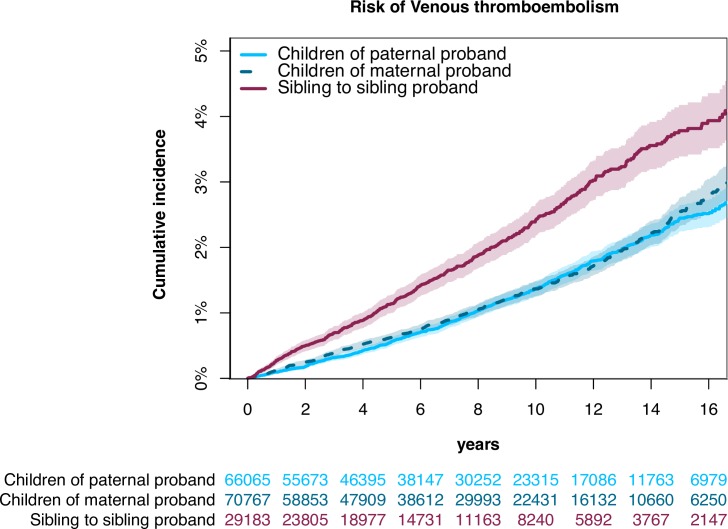
Cumulative incidence of VTE among first-degree relatives. Absolute risk of VTE among first-degree relatives to patients previously hospitalized with VTE. The numbers below the figure represents the number of patients still at risk, i.e. patients who are still in the study.

**Fig 3:**
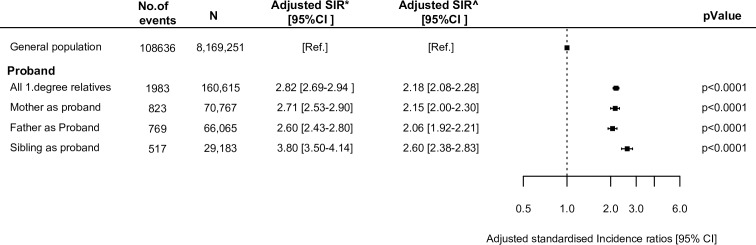
Standardized incidence ratios (SIRS) of VTE among first-degree relatives. Relative rate of VTE among first-degree relatives to patients with VTE. The reference is the general population. CI = confidence interval. *Adjusted for age, sex and calendar year. ^Adjusted for age, sex, calendar year, comorbidities, and concomitant medication.

Fig 2 shows the cumulative incidence of VTE among first-degree relatives. At 16 years the cumulative incidence was 2.5% [95% CI: 2.3–2.8%], 2.4% [95%CI: 2.2-2-6%], and 3.8% [95% CI: 3.4-4-2%] among children with maternal, paternal, and sibling-to-sibling probands, respectively. A significantly higher cumulative incidence was observed among siblings-to-sibling probands compared with children of maternal and paternal probands. The relative rate of VTE was significantly increased among all first-degree relatives compared with the general population and with the highest estimates found among siblings (SIR of 2.60 [95% CI: 2.38–2.83], Fig 3).

**Fig 4:**
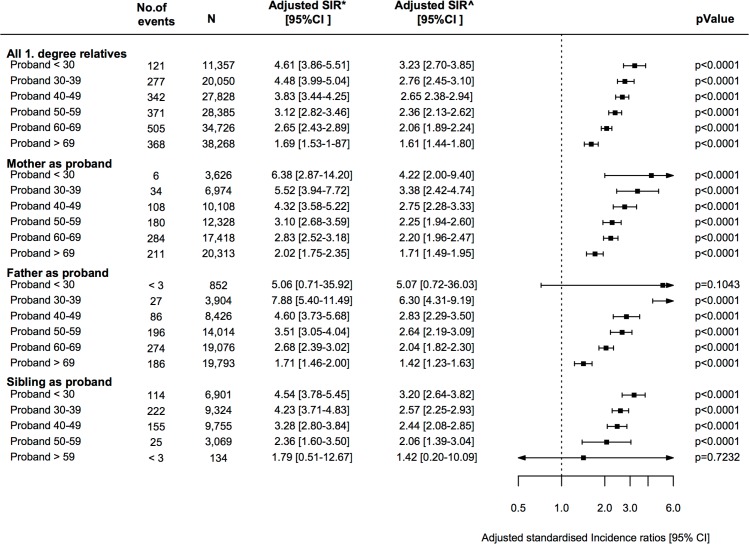
Standardized incidence ratios (SIRS) of VTE among first-degree relatives. Relative rate of VTE among first-degree relatives to patients with VTE. The risk is shown according to the age of diagnosis of the proband, here divided into age groups of the proband. The reference is the general population. CI = confidence interval. *Adjusted for age, sex and calendar year. ^Adjusted for age, sex, calendar year, comorbidities, and concomitant medication.

Furthermore a “dose-response” relationship was observed; the lower the age at diagnosis of the proband, the higher rate of VTE was observed among first-degree relatives (p-value for interaction with age < 0.001) (Fig 4). Further, the relative rate of VTE was significantly increased when the proband was diagnosed at a young age (≤ 50 years of age, p-value for interaction<0.001) (S3 Table).

### Sensitivity analyses

**Fig 5:**
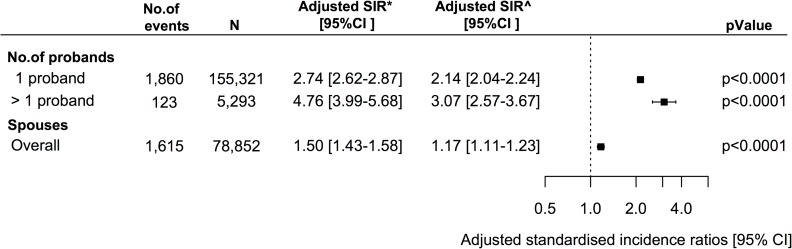
Standardized incidence ratios (SIR) of VTE among first-degree relatives according to number of probands and among spouses. Relative rate of VTE among first-degree relatives according to number of probands and among spouses. The reference is the general population. CI = confidence interval. * Adjusted for age, sex and calendar year. ^Adjusted for age, sex, calendar year, comorbidities, and concomitant medication.

In an analysis exploring the rate of VTE in first-degree relatives with one or several probands previously hospitalized with VTE the highest estimate was observed among first-degree relatives with more than one VTE proband (Fig 5). Lastly, examining the rate of VTE among spouses revealed a significantly increased relative rate of VTE among spouses compared with the general population (Fig 5).

## Discussion

In this nationwide cohort-study covering a thirty-four year time period, we investigated the rate of VTE among first-degree relatives to individuals previously diagnosed with VTE. Our main findings revealed a significantly increased rate of VTE among first-degree relatives compared with the Danish background population irrespective of whether the proband was a mother, father, or a sibling albeit with the highest estimates found among siblings. Noteworthy, when the proband had VTE in a young age the relative rates of VTE in first-degree relatives were significantly higher.

### Findings in relation to other studies

Results from this study, is consistent with those reported from other studies, that a family history of VTE is associated with a two- to threefold increased risk of VTE in first-degree relatives, with the risk increasing according to age at diagnosis for the proband and number of affected probands. [17, 19, 20, 22, 29, 30, 37] However, some of these studies have been small, and have been prone to recall and selection bias. [17, 22, 29] Zoller et al, have conducted several large cohort studies on a Swedish population, but these studies have been limited by the lack of information on, and control for, important risk factors for VTE. [19, 20, 30]

Similarly to Zoller et al, we observed an increased risk of VTE when the proband was a father, mother or a sibling. [19, 30] Interestingly, the highest relative risk of VTE was observed when the proband was a sibling. This association has not been described previously, and can possibly be explained by siblings sharing more of the same environmental risk factors. Another important observation was that the highest estimates were observed among first-degree relatives with more than one proband, regardless of type of probandship. Other studies have shown a similar trend [17–20], which emphasizes the importance of considering the highly increased risk associated with having more than one proband.

Additionally, we observed a reverse relationship between the rate of VTE and age at VTE diagnosis for the proband, other studies have reported a similar trend, and this may advocate for a genetic component. [20, 21, 30] The relationship between age at diagnosis of the proband, and the increased rate observed among first-degree relatives with more than one affected proband, suggests that the disease follow complex traits, rather than following classical Mendelian genetics with monogenic traits, possibly caused by an additive genetic effect or by gene-gene interactions. [19, 38, 39] As of today, 17 genes have been associated with genetic variations increasing the risk of VTE. [9] However, common polymorphisms have been estimated to only account for 5% of VTE heritability [9] and thus further studies on common and especially rare variants are needed to disentangle the genetic structure of VTE heritability. [9]

Zoller et al. investigated the familial risk of VTE among adoptees, and found that adoptees with affected non-biological parents did not have a significantly increased risk of VTE,.[40] However, it was found that adoptees with affected biological parents had a significantly increased risk of VTE.[40] The study suggested that genetic factors contributed more to the risk of VTE than environmental factors.[40] We observed lower estimates of VTE risk among spouses than among first-degree relatives as compared with the general population, suggesting that the genetic component plays a large role compared with environmental factors.[40] To further investigate to which extend familial environment influences the rate of VTE, we aimed at performing an analysis of the rate of VTE among adopted children with adoptive parents having VTE. However, due to a sparse number of events this analysis was not feasible.

### Clinical implications

Results from this study will help physicians in identifying high-risk patients by assessing family history of venous thrombosis. It is important for the clinical practice to be able to identify high-risk patients that could potentially benefit primary prophylactic anticoagulation treatment in situations that are under normal circumstances considered low risk [8, 41]. However, it is unknown in which situations primary prophylaxis can be justified, and further research is needed to clarify this. Secondary prophylaxis can however not be justified, as a family history with VTE has not been associated with an increased risk of recurrent VTE. [8]

### Strengths and limitations

Family aggregation studies provides the basis for the investigation of both genetic and environmental risk factors in families [37]. By using the Danish nationwide registries, it was possible to include an unselected cohort of patients, with a great variety of comorbidities. [31, 42] It was possible to include a large study population, which provided robust estimates for the relative risks. Furthermore, the registration of variables in the utilized registries has been associated with high validity. [31–33, 42] As of limitations, the diagnosis of VTE has been associated with a positive predictive value of 75%, however this misclassification is likely the same for all groups being investigated. [43] Another limitation to this study is the lack of information on certain risk factors for VTE, such as known thrombophilia, BMI, smoking, use of oral contraceptives, use of hormonal therapy, and alcohol use, which were not readily available from the registries. Thus it was not possible to control for these potential confounders. Lastly, because of the observational study design, this study is based on associations and thus cannot comment on the causal relationship between family history and risk of VTE. Furthermore, the influence on social heritability was not investigated, which provides basis for future research.

## Conclusion

This study is one of the largest cohort studies to describe the familial clustering of VTE. It was found that a family history of VTE was associated with an increased rate of VTE among first-degree relatives. The highest rates were observed when the proband was a sibling, when having more than one proband and when the proband was diagnosed with VTE in a young age. Results from this study have provided robust estimates for the risk of VTE among first-degree relatives, and emphasizes the importance of considering family history of VTE when identifying high-risk patients.

## Supporting Information

S1 Table List of ICD-8 and 10 diagnosis codes for diagnoses (DOCX)

S2 Table ATC-codes for concomitant medication (DOCX)

S3 Table Standardized incidence ratios (SIRS) of VTE according to age of at diagnosis for proband (DOCX)
