# Supplementary material for: Familial Clustering of Venous Thromboembolism – A Danish Nationwide Cohort Study
Source: PLoS One. 2016 Dec 29;11(12):e0169055. doi: 10.1371/journal.pone.0169055 (PMC5199093; doi:10.1371/journal.pone.0169055)
Supplement: S1 Table — (DOCX) [file pone.0169055.s001.docx]

## S1 Table: List of ICD-8 and 10 diagnosis codes for diagnoses

| List of variables |  |  |
| --- | --- | --- |
| *Venous thromboembolism*  *(A+B diagnoses, in patient and out-patients diagnoses)* | | |
| *Pulmonary embolism* | *Defined from diagnosis* | ICD-10: DI26  ICD-8: 45099 |
| *Deep venous thrombosis* | *Defined from diagnosis* | ICD-10: DI80, DI821, DI822, DI823, DI828, DI829  ICD-8: 451-453 |
|  |  |  |
| Comorbidities  *(A+B diagnoses, patient type= 0 and 2)* | | |
| *Ischemic heart disease* | *Defined from diagnosis* | ICD-10: DI20-25  ICD-8: 410-414 |
| *Vascular disease* | *Defined from diagnosis* | ICD-10: DI70  ICD-8: 440 |
| *Arterial embolism* | *Defined from diagnosis* | ICD-10: DI74  ICD-8: 444 |
| *Acute myocardial infarction* | *Defined from diagnosis* | ICD-10: DI21, DI22  ICD-8: 410 |
| *Stroke* | *Defined from diagnosis* | ICD-10: DI63, DI64  ICD-8: 433-434 + 43601 + 43690 |
| *Atrial fibrillation* | *Defined from diagnosis* | ICD-10: DI48  ICD-8: 427930, 427940 |
| *Cancer* | *Defined from diagnosis* | ICD-10: DC  ICD-8: 140-209 |
| *Chronic kidney disease* | *Defined from diagnosis* | ICD-10: DE102, DE112, DE132, DE142, DI120, DN02, DN03, DNO4, DN05, DN06, DN07, DN08, DN11, DN12, DN14, DN158, DN159, DN160, DN162 , DNI63, DN164, DN168, DN18, DN19, DN26, DQ61, DM300, DM313, DM319, DM321B  ICD-8: 582-584, 24902, 40399, 58000,581, 59009, 591 |
|  |  |  |
| *Abnormal liver function* | *Defined from diagnosis* | DB15, DB16, DB17, DB18, DB19, DC22, DD684C, DK70, DK71, DK72, DK73, DK74, DK75, DK76, DK77, DZ944, DQ618A  ICD-8: 570 |
| *Chronic obstructive pulmonary disase* | *Defined from diagnosis* | ICD-10: DJ42, DJ43, DJ44  ICD-8: 490-492 |
|  |  |  |
| *Chronic heart failure* | *Defined from diagnosis* | ICD-10: DI110, DI42, DI50, DJ81  ICD-8: 427 |
|  |  |  |
| *Diabetes* | *Defined from diagnosis* | ICD-10: E10, E11, E12, E13, E14  ICD-8: 250 |
|  |  |  |
