# Supplementary material for: Familial Clustering of Venous Thromboembolism – A Danish Nationwide Cohort Study
Source: PLoS One. 2016 Dec 29;11(12):e0169055. doi: 10.1371/journal.pone.0169055 (PMC5199093; doi:10.1371/journal.pone.0169055)
Supplement: S2 Table — (DOCX) [file pone.0169055.s002.docx]

## S2 Table : ATC-codes for concomitant medication

| Concomitant medication |  | ATC-codes: |
| --- | --- | --- |
| *ADP-receptor blockers* | Defined from ATC-codes | B01AC except B01AC06/ B01AC56 |
| *Aspirin* | Defined from treatment with acetylsalicylic acid | B01AC06, N02BA01, B01AC56 |
| *Ulcus medication* | Defined from ATC-codes | A02 |
| *Lipid modifying drugs* | Defined from ATC-codes | C10 |
| *Non steroidal anti-inflammatory drugs* | Defined from ATC-codes | M01A except M01AX05 |
| *Oral anticoagulation therapy* |  | B01AA, B01AA03, B01AA04, B01AE07, B01AF01, B01AF02, B01AX06 |
| *Heparin* | Defined from ATC-codes | B01AB |
| *Digoxin* | Defined from ATC-codes | C01A |
| *Diuretics* | Defined from ATC-codes | C02L, C03A, C03B, C03D, C03E, C03X, C07C, C07D, C08G, C02DA, C09BA, C09DA, C09xa52 |
| *Loop* | Defined from ATC-codes | C03C |
| *Diuretics+ other antihypertensive drugs* | Defined from ATC-codes | C02L: |
| *Beta- blocking agents* | Defined from ATC-codes | C07A |
|  |  |  |
| *Beta blocking agents combined with diuretics* | Defined from ATC-codes | C07B , C07C, C07D |
| *Beta blocking agents combined with calcium channel antagonists* | Defined from ATC-codes | C07F |
| *Calcium channel antagonists* | Defined from ATC-codes | C08 |
| *Calcium channel antagonists and diuretics* | Defined from ATC-codes | C08G |
| *Renin angiotensin system inhibitors* | Defined from ATC-codes | C09XA02 |
| *Renin angiotensin system inhibitors + diuretics* | Defined from ATC-codes | C09BA, C09DA |
|  |  |  |
| *Renin angiotensin system inhibitors + calcium channel blockers* | Defined from ATC-codes | C09BB, C09DB |
| *Antipsychotics* | Defined from ATC-codes | N05A |
| Anti-diabetics without insulin | Defined from ATC-codes | MA10B |
| Anti-diabetics with insulin | Defined from ATC-codes | MA10A |
