# Supplementary material for: Familial Clustering of Venous Thromboembolism – A Danish Nationwide Cohort Study
Source: PLoS One. 2016 Dec 29;11(12):e0169055. doi: 10.1371/journal.pone.0169055 (PMC5199093; doi:10.1371/journal.pone.0169055)
Supplement: S3 Table — (DOCX) [file pone.0169055.s003.docx]

## S3 Table: Standardized incidence ratios (SIRS) of VTE according to age of at diagnosis for proband

|  | **No. of patients** | **No. of events** | **Unadjusted RR** | **Adjusted RR** |
| --- | --- | --- | --- | --- |
| **All first degree relatives** | |  |  |  |
| Proband < 50 | 59,235 | 740 | 4.17 [3.88-4.82] | 4.61 [3.93-5.42] |
| Proband > 50 | 101,379 | 1243 | 2.36 [2.31-2.49] | 1.93 [1.83-2.04] |
| **Children of maternal probands** | |  |  |  |
| Proband < 50 | 20,708 | 148 | 4.61 [3.92-5.42] | 2.92 [2.49-3.43] |
| Proband > 50 | 50,059 | 675 | 2.48 [2.30-2.68] | 2.03 [1.88-2.19] |
| **Children of paternal probands** | |  |  |  |
| Proband < 50 | 13,182 | 144 | 5.11 [4.25-6.14] | 3.27 [2.72-3.93] |
| Proband > 50 | 52,833 | 655 | 2.40 [2.23-2.59] | 1.93 [1.79-2.09] |
| **Sibling to sibling probands** | |  |  |  |
| Proband < 50 | 25,980 | 491 | 3.93 [3.60-4.30] | 2.64 [2.42-2.88] |
| Proband > 50 | 3,203 | 26 | 2.33 [1.59-3.34] | 2.02 [1.38-2.97] |
